# A single-molecule assessment of the protective effect of DMSO against DNA double-strand breaks induced by photo-and γ-ray-irradiation, and freezing

**DOI:** 10.1038/s41598-017-08894-y

**Published:** 2017-08-17

**Authors:** Masami Noda, Yue Ma, Yuko Yoshikawa, Tadayuki Imanaka, Toshiaki Mori, Masakazu Furuta, Tatsuaki Tsuruyama, Kenichi Yoshikawa

**Affiliations:** 1 Faculty of Life and Medical Sciences, Doshisha University, Kyoto, 610-0321 Japan; 2 Research Organization of Science and Technology, Ritsumeikan University, Shiga, 525-8577 Japan; 3 Graduate School of Engineering, Department of Quantum and Radiation Technology, Osaka Prefecture University, Osaka, 599-8570 Japan; 4 Drug Discovery and Medicine, Department of Pathology, Graduate School of Medicine, Kyoto University, Kyoto, 606-8501 Japan

## Abstract

Dimethyl sulfoxide (DMSO) is widely used as a cryoprotectant for organs, tissues, and cell suspension in storage. In addition, DMSO is known to be a useful free radical scavenger and a radio-protectant. To date, many *in vitro* assays using cultured cells have been performed for analysing the protective effect of DMSO against genomic DNA damage; however, currently it has been rather difficult to detect DNA double strand breaks (DSBs) in a quantitative manner. In the present study, we aimed to observe the extent of DNA damage by use of single molecular observation with a fluorescence microscope to evaluate DSBs induced by photo- and γ-ray-irradiation, or freeze/thawing in variable concentrations of DMSO. As a result, we found that 2% DMSO conferred the maximum protective effect against all of the injury sources tested, and these effects were maintained at higher concentrations. Further, DMSO showed a significantly higher protective effect against freezing-induced damage than against photo- and γ-ray-irradiation-induced damage. Our study provides significant data for the optimization of DNA cryopreservation with DMSO, as well as for the usage of DNA as the protective agent against the injuries caused by active oxygen and radiations.

## Introduction

Increasing evidence suggests that genomic DNA damages induced by various endogenous or environmental factors. Environmental damage is caused by agents such as ultraviolet, X-ray, and γ-ray irradiations; thermal disruption; aromatic intercalating compounds; and viral infection. Compared to other types of DNA damage, such as 8-hydroxydeoxyguanosine residues and polycyclic aromatic hydrocarbon adducts and single-strand breaks, double-strand breaks (DSBs) are regarded as most serious because they lead to cancer and cell-death^1–7^.

Many *in vivo* and *in vitro* studies have been conducted to detect DSBs. Polymerase chain reaction (PCR) can be used to assess DNA damage by detection of amplification termination^8^. Immunological assays are also frequently used to detect oxidative DNA damage by using an antibody against damaged DNA^8, 9^. *In situ* hybridization assay, using a probe for certain DNA sequences, provides information on nucleotide changes^8^. The comet assay also detects DSBs^8, 10^. Despite the availability of these methods, it has been difficult to quantitatively evaluate the number of DSBs at a single-molecule level, particularly in case of long genome-sized DNA strands. Recently, the direct visualization of single DNA molecules by fluorescence microscopy for quantitatively analysing DSBs in genome-sized DNA strands^11–13^ has been demonstrated^14–21^.

Dimethyl sulfoxide (DMSO) is known to be a useful free radical scavenger and a radio-protectant^7, 22–28^. In fact, it has been reported that DMSO reduces the degree of radiation injury of adjacent organs in cancer radiotherapy. Radiation damage can be classified as direct or indirect^26, 29^. In the indirect mechanism, the irradiation of organs as well as the cellular medium causes formation of chemically active species, i.e., reactive oxygen species (ROS), such as the hydroxyl radical and methyl radical^29, 30^. However, the mechanism underlying chemical reduction of reactive species by DMSO is still unclear^22^. DMSO is one of the most important agents in cryopreservation, i.e., it protects living cells, organs, and tissues during storage at freezing temperatures^22, 23, 31–40^. When the DSBs occur in the preserved cells, the damage is hazardous to the cell, and the viability after preservation will be significantly lower, because the cell will not survive during subsequent cell mitosis after thawing. This protective effect on DNA has been argued to be related to the strong solvation effect of DMSO on water molecules^23, 31–33, 41^. DMSO interacts with water molecules through the two hydrogen bonds of water^40^.

Despite these useful practical applications of DMSO, the protective effect of DMSO against DSBs induced due to radiation or freezing/thawing has not yet been evaluated quantitatively. In the present study, in order to quantitatively evaluate the protective effects of DMSO against DSBs on genomic DNA molecules by use of single DNA observation, we induced DSBs by several different injury sources; photo-induced reactive oxygen, γ-ray irradiation, and freeze/thawing. To observe the effect of photo-induced ROS, YOYO-1, a fluorescent cyanine dye, was used as a photosensitizer to generate ROS^42, 43^, and the real-time observation of DSBs in individual DNA molecules was performed, where YOYO-1 also helps to visualise DNA under visible light^44–47^. With regard to γ-ray irradiation, the number of DSBs was evaluated in terms of the average length of DNA molecules at different degrees of irradiation in the presence of DMSO. Likewise, we also evaluated the degree of DSBs caused by freezing/thawing, with two cold temperatures (−25 °C and −80 °C), by considering the phase boundary of freezing in a water-DMSO solution^38^. Because thermal disruption is one of the critical factors in cryopreservation, application of our method will provide a critical knowledge about freezing/thawing stress on preserved DNA.

## Results

### Protective effect of DMSO against DSBs caused by photo-irradiation-induced ROS

**Figure 1:**
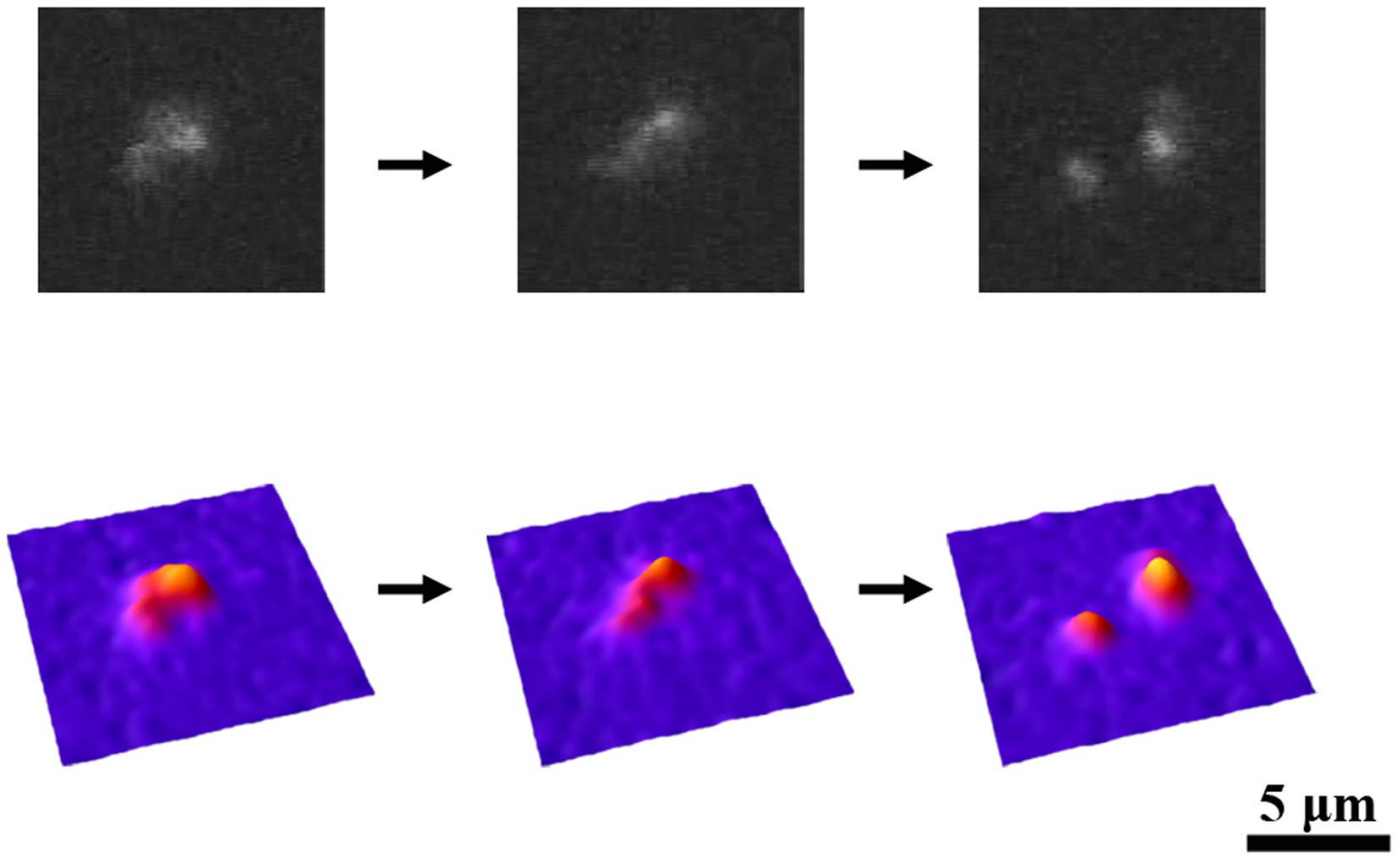
Example of the real-time observation of DSB caused by photo-irradiation-induced ROS. Fluorescence microscopic images of a single T4 DNA molecule under photo-irradiation (upper), and the corresponding quasi-three-dimensional profiles of the fluorescence intensity distribution (bottom). (Fluorescence dye: 0.05 μM YOYO-1).

We measured the breakage time, τ of individual DNA molecules in solution under focused illumination by fluorescence microscopy. Figure 1 exemplifies the real-time observation of the breakage of a single T4 DNA molecule, indicating that the breakage reaction is actually observed at the level of individual DNA molecules. On the measurements, YOYO-1 was adapted as a photosensitizer to generate ROS and also used as a fluorescence dye to visualize DNA molecules. The corresponding quasi-three-dimensional images of the individual photo were acquired for illustration of the fluorescence intensity distribution for the DNA molecule (bottom images, Fig. 1). Thus, the time-successive observation with fluorescence microscopy made it possible to monitor the process of double-strand breakage in individual molecules. The breakage time, τ, was evaluated for the period from the moment of the start of focused illumination until the first double-strand breakage.

**Figure 2:**
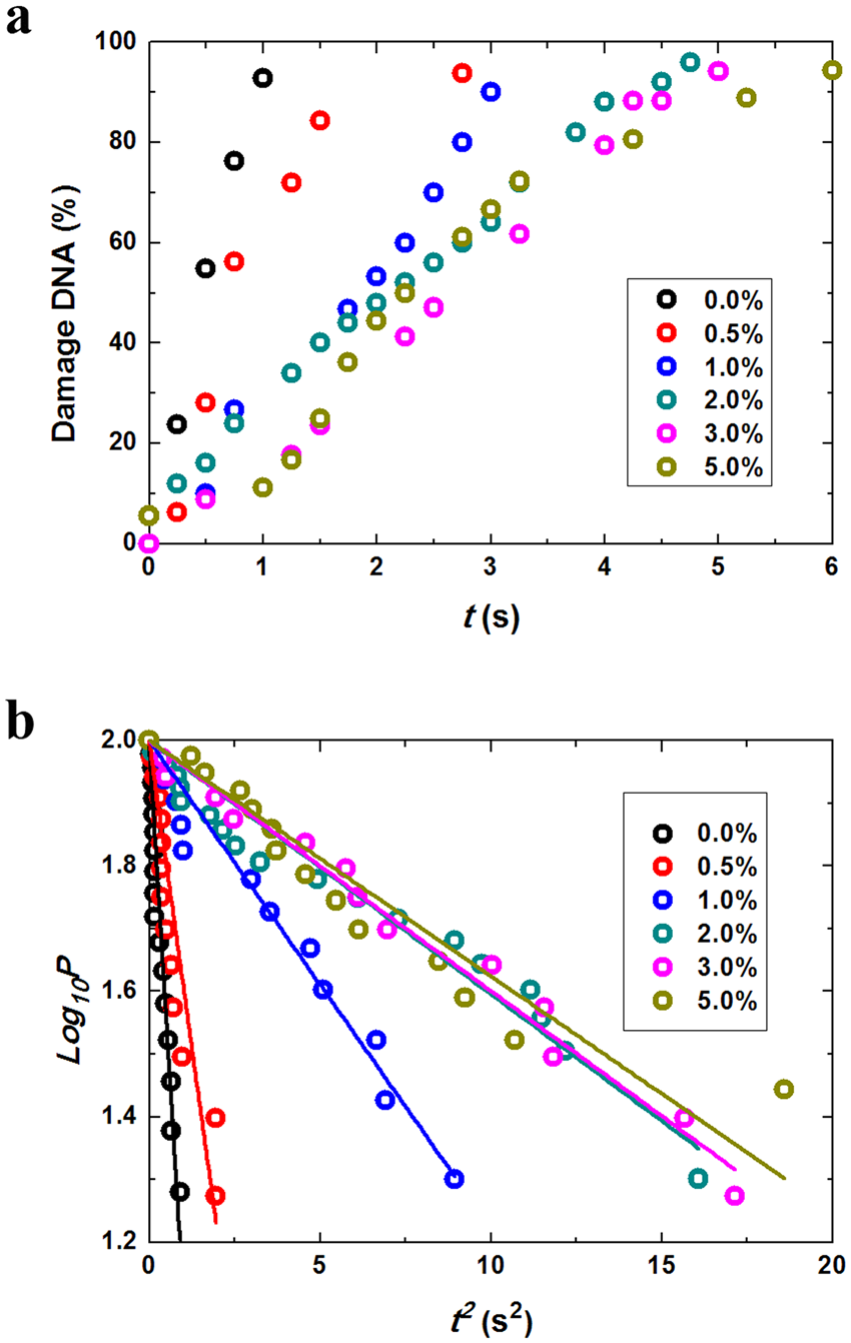
Photo-induced DSBs. (**a**) Time-dependence of the percentage of damaged DNA molecules at different DMSO concentrations. (**b**) The relationship between *t* ^2^ and log_10_ *P*, where *P* is the percentage of surviving DNA molecules, which was calculated as [100% − (percentage of damaged DNA)]. (The kinetic constants, K _v_’s (s^−2^), are evaluated from the slopes of Fig. 2b. For DMSO’s concentration on 2%, 3% and 5%, the slopes are essentially the same within experimental errors, indicating the presence of saturation effect).

Subsequently, a time-dependent increase in photo-irradiation-induced DNA damage in the solution containing different concentrations of DMSO was evaluated by calculating the percentage of damaged DNA molecules through double-strand break relative to the total DNA molecules (Fig. 2a **)**, where the numbers of observed DNA molecules at each concentration of DNA were 50–100. In Fig. 2b, the vertical axis is the logarithm of the probability of surviving DNA, *P*; where [*P* (%)] + [damaged DNA (%)] = 100%. The horizontal axis is the square of the irradiation duration, *t* ^2^. The linear correlation between the square of breakage time and log_10_ *P* in Fig. 2b suggests that the kinetics of double-strand breakage is given as the product of two independent events, i.e., DSBs are induced via a two-step mechanism as described below. In case of single-strand breaks (SSBs), nicks are generated randomly along the double-stranded DNA molecules under irradiation, and fragmentation of a DNA molecule is induced by an additional SSB near an existing SSB^42^. We previously reported the details of the kinetics of DSBs through a similar two-step mechanism^15, 44^. Under constant illumination with a power *I*, the number of nicks along a single DNA molecule will increase as shown in equation (1), where *α* is a positive constant:

1 $$dn/dt=\alpha I$$

after integration, by setting *n* (number of nicks) = 0 at *t* = 0, we obtain:

2 $$n=\alpha It$$

By denoting *P* as the probability (percentage) of surviving DNA molecules against double-strand damage, the rate of the decrease in *P* can be represented as the product of *n* and *P*:

3 $$dP/dt=-knP=-k\alpha ItP$$

where *k* is the rate constant. Thus, we obtain:

4 $${\mathrm{log}}_{10}(P/{P}_{0})\propto -\alpha I{t}^{2}$$

and by introducing an initial constant of *P*_0_ = 1 at *t* = 0, we obtain:

5 $${\mathrm{log}}_{10}P=-{K}_{v}{t}^{2}$$

where *K*_*v*_ is the rescaled kinetic constant. The linear relationships between the square of the time and log_10_ *P* in Fig. 2b confirm that the above-mentioned two-step reactions define the underlying mechanisms of DSBs caused by photo-induced ROS. From Fig. 2a and b, it was apparent that the protective effect increased with an increase in DMSO concentration and this effect was essentially maintained in DMSO concentrations higher than 2%. From the slope of the graph in Fig. 2b, the relative kinetic constant of the DSB reaction was deduced to be, *k* = *K*_*v*_/*K*_*v*_^0^, where *K*_*v*_^0^ is the constant in the absence of DMSO. The relative kinetic constant, *k*, at different DMSO concentrations was calculated from the slope in Fig. 2b.

### Protective effect of DMSO against γ-ray-induced DSBs

**Figure 3:**
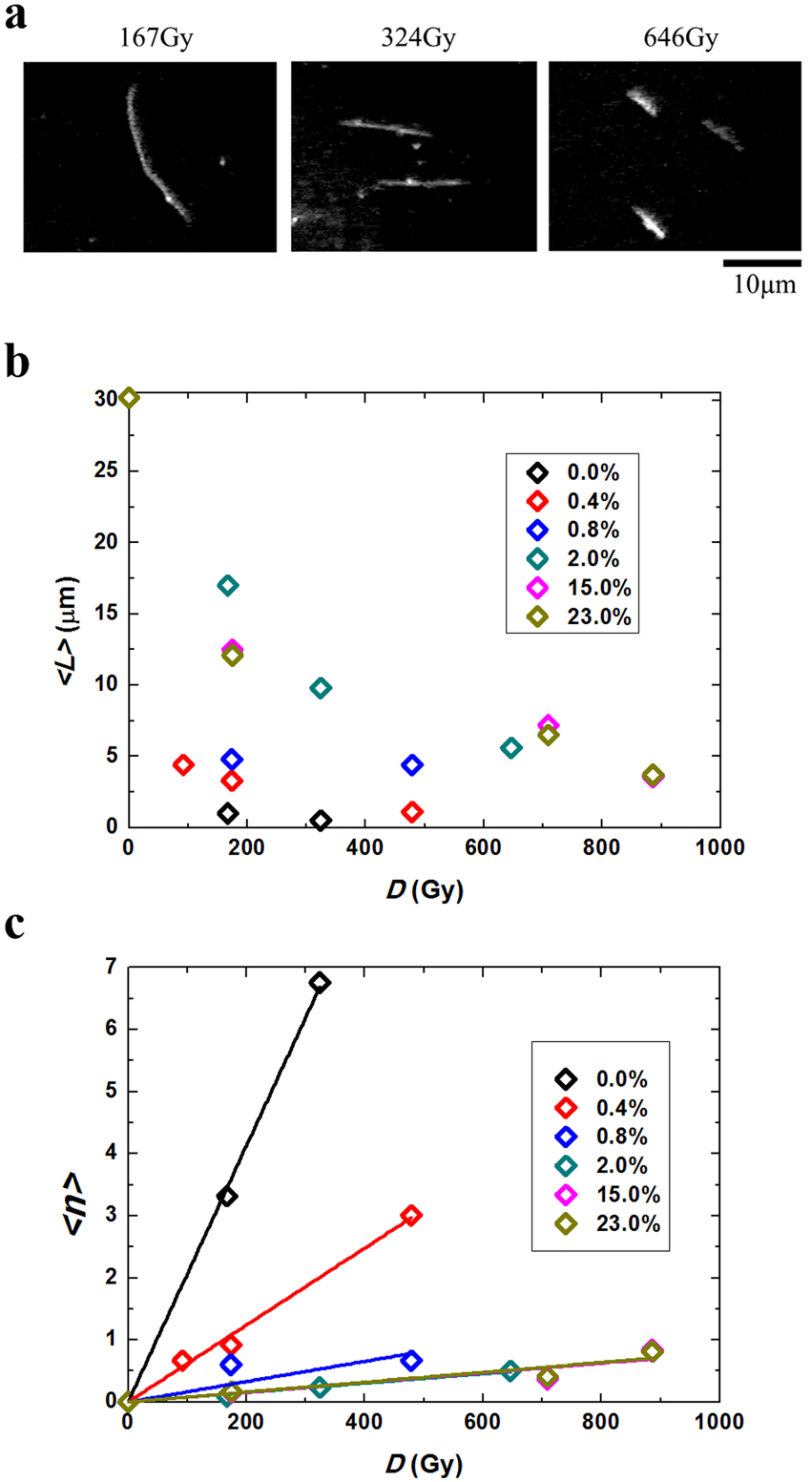
DSBs induced by γ-ray. (**a**) Fluorescence microscopic images of DNA molecules fixed on a glass surface after irradiation with different doses of γ-ray. (**b**) Average DNA lengths, 〈*L*〉, vs. the irradiation dose of γ-rays. (**c**) Number of DSBs per 10 kbp, 〈*n*〉, vs. the irradiation dose of γ-rays. (The kinetic constants, *Kγ* ‘s (Gy), are evaluated from the slopes of Fig. 3c.) The slopes are essentially the same for the DMSO concentrations above 2%, indicating saturation effect.

Fluorescence images of the T4 DNA fixed on a glass substrate after variable γ-ray-irradiation doses (Gy) are exemplified in Fig. 3a, indicating that the length of DNA molecules decreased with a higher level of γ-ray-irradiation. The average length of DNA in a single observation, 〈*L*_0_〉, was determined to be 30.2 μm for the control sample before γ-ray-irradiation. For the target DNA strands, 〈*L*〉 was plotted for the irradiation doses (Fig. 3b), where the average length was evaluated based on the length distributions of the samples corresponding to ca.50 control DNA molecules at each DMSO concentration. The average number from the data for 〈*L*〉 and DSBs per individual DNA molecule, 〈*N*_*r*_〉, were evaluated by using the following equation^15, 44^:

6 $$\langle {N}_{{\rm{\gamma }}}\rangle =\langle {L}_{0}\rangle /\langle L\rangle -1$$

To compare the number of DSBs in a quantitative manner, a new parameter 〈*n*〉, the average number of DSBs per 10 kbp was introduced:

7 $$\langle n\rangle =\langle {N}_{\gamma }\rangle \cdot 10/{X}_{0}$$

Here, *X*_0_ is represented by the unit of kbp for DNA under control conditions, which is deduced by comparison with the natural contour length (57 μm, 166 kbp).

8 $${X}_{0}=(\langle {L}_{0}\rangle \cdot 166)/57$$

As a result, the data of a linear correlation between 〈*n*〉 and irradiation dose, *D*, was obtained, suggesting that the increase in DSBs was proportional to the dose of γ-ray-irradiation (Fig. 3c). This linearity implies that the DSBs induced by γ-ray are caused in a single-step reaction, i.e., one DSB is caused by a single γ-ray photon. In contrast, in the ROS attack emitted from the fluorescence dye under visible-light irradiation, one DSB was caused by a pair of neighbouring SSBs on complementary strands^15, 44^. In this figure, the slope, *K*_*γ*_ represents the kinetic constant under the framework of a one-step reaction mechanism. As shown in Fig. 3c, *K*_*γ*_ decreases monotonously with an increase in DMSO concentration up to 2% and was kept almost constant with a further increase in the concentration. Thus, the protective effect reached a plateau at 2% DMSO concentration.

### Protective effect of DMSO against freezing-induced DSBs

**Figure 4:**
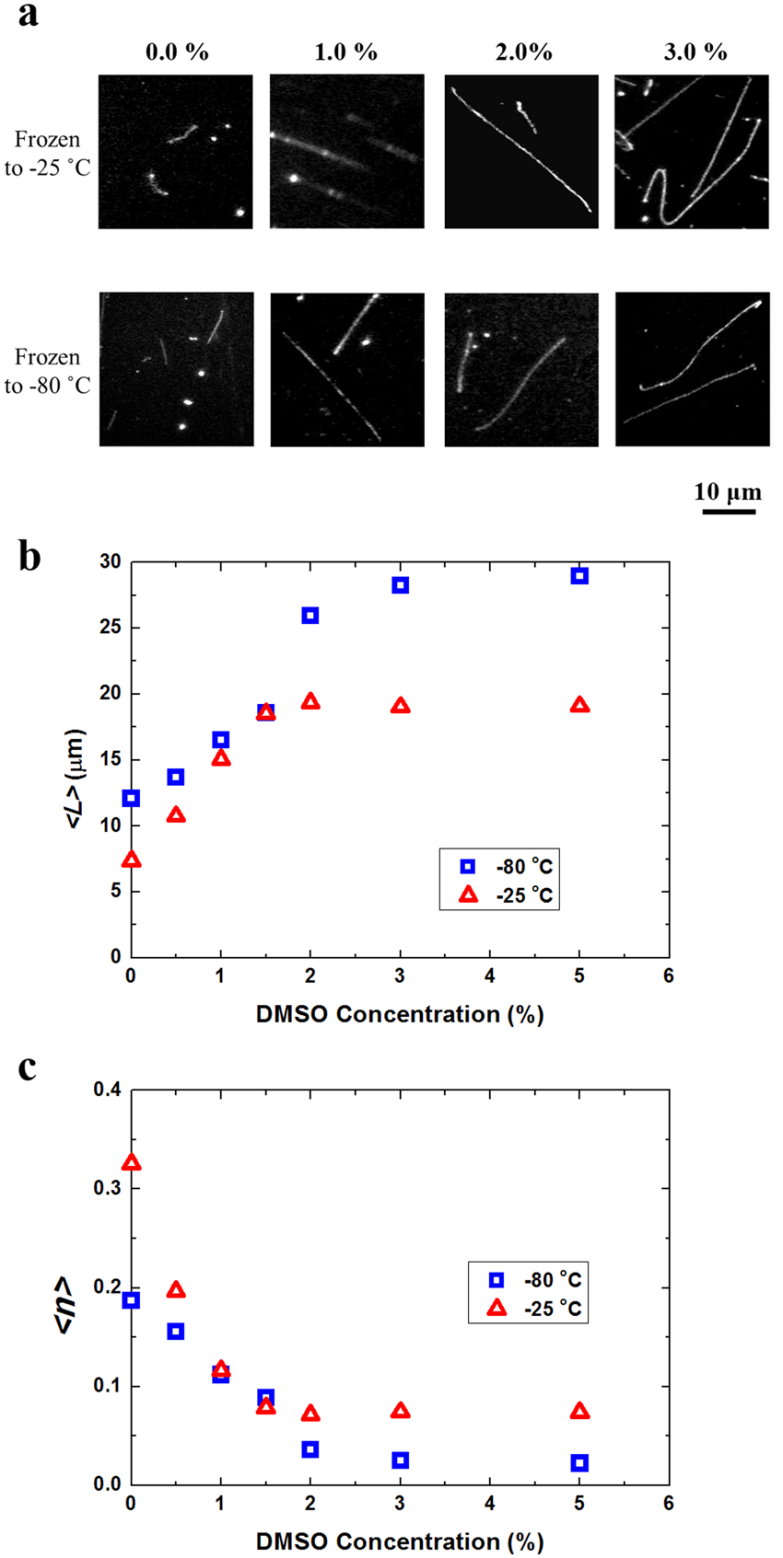
Freezing-induced DSBs. (**a**) Single DNA image after freeze/thawing to −25 °C (upper: slow frozen) and −80 °C (lower: quick frozen). (**b**) Average lengths of DNA, 〈*L*〉, vs. the concentration of DMSO. (**c**) The number of DSBs per 10 kbp, 〈*n*_*f*_〉, vs. the concentration of DMSO.

Finally, DSBs caused by freeze/thawing in the solution containing variable DMSO concentrations were assessed by fluorescence microscopy using the DNA fixed on a solid substrate (Fig. 4a). Further, the average length at different DMSO concentrations for freezing at −25 °C (slow frozen) and −80 °C (quick-frozen) were measured (Fig. 4b). Similar to the procedure on the evaluation of the average length for the experiments by γ-ray-induced DSBs, we have measured the length distributions of the samples corresponding to 30–50 control DNA molecules at each conditions. By using a method similar to that used to assess the γ-ray-induced DSBs, the relationship between the number of DSBs per 10 kbp, 〈*n*_*f*_〉, and the DMSO concentration was obtained (Fig. 4c). As shown, the probability of DSBs was higher for freezing to −25 °C than for quick freezing to −80 °C^48^. DMSO was found to have a protective effect against DSBs and significantly, this effect reached a plateau at 2% DMSO. Like the protective effects of DMSO against damage caused by photo-induced ROS and γ-ray-irradiation, the protective effect of DMSO against freezing-induced damage was maximum at a 2% concentration. Here, it is noted that the effect of freezing point depression with 2% DMSO is rather small, i.e., on the order of −1 degree^49^. Nontheless, we may expect large difference of the kinetics of ice-formation even at such low concentration of DMSO.

## Discussion

In the current study, we induced DSBs in the target DNA using different sources and assessed DSBs directly by observing under a fluorescent microscope. The data provided the common kinetics of DSB formation by the variable resources.

**Figure 5:**
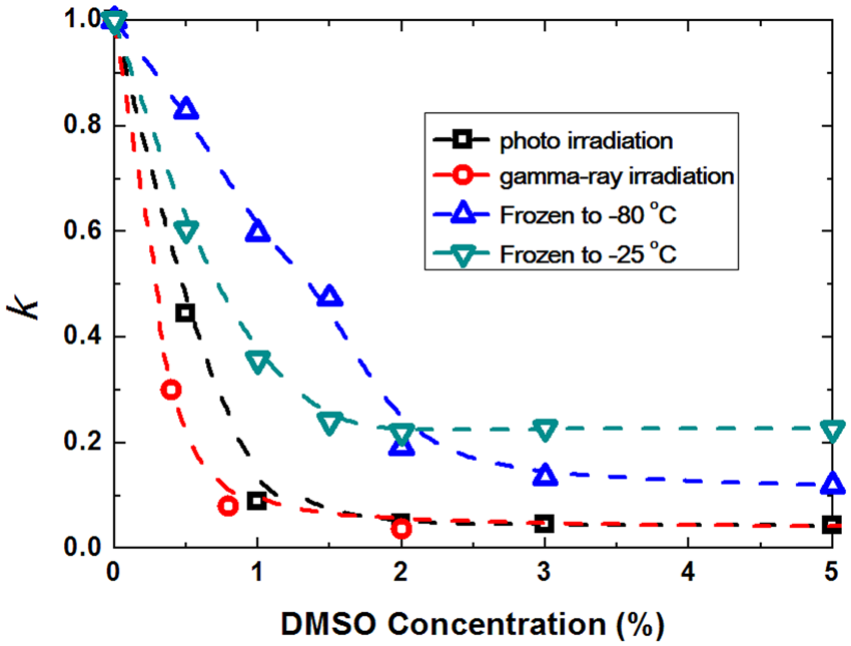
Difference in the protective effect of DMSO. Vertical axis is the relative kinetic constant *k* = *K*/*K*_0_ for the generation of DSBs at different concentrations of DMSO, where *K*_0_ is the rate constant in the absence of DMSO. With respect to freezing, ‘quick freezing’ is to −80 °C, and ‘slow freezing’ is to −25 °C.

To compare the protective effects of DMSO against DSBs, caused due to damage by different sources, based on experimentally available kinetic constants, *K*_*v*_, *K*_*γ*_, and *K*_*f*_: *n*_*f*_, we introduced a relative constant, *k* = *K/K*_0_, where *K*_0_ is the kinetic constant of the control group for each injury source in the absence of DMSO. Using this renormalized constant *k*, it is possible to compare the degree of the protective effect of DMSO, regardless of any difference in the mechanism of double-strand breakage, including whether the mechanism involves either one-step or two-step reactions. Changes in the relative kinetic constant, *k*, under different concentrations of DMSO are summarised in Fig. 5. The DSBs caused by photo- and γ-ray-irradiation significantly decrease to the order of 1/100 when the concentration of DMSO is above 1.5–2.0%. In contrast, the decrease in the rate constant of DSBs remains in the order of 1/5–1/10 for injury due to freeze/thawing, even at a DMSO concentration >2%. This large difference in protection is attributable to the difference in the physical-chemical mechanism of DSBs. Considering freezing, the growth of ice crystals is considered to be the main cause of DSBs^50–52^. When DMSO is added to an aqueous solution, the ice crystallization is expected to reduce, and thus the double-strand breakage would be decreased. On the contrary, γ-ray-irradiation is considered to cause DSBs mainly through an indirect mechanism, i.e., generated ROS may attack DNA molecules. It has been reported that a single γ-ray photon can produce several reactive species, which produce a DSB in a one-step process. For damage caused by photo-irradiation, DSBs are induced mainly through a two-step mechanism. Interestingly, DMSO has a strong protective effect against ROS, regardless of whether the mechanism is single-step or two-step.

The protective effects of DMSO against photo- and γ-ray irradiations were about 1.2-fold less than that against freeze-thawing. This difference between radical protection and freezing protection is attributable to the different mechanisms of radical damage (photo- and γ-ray-irradiation) and physical damage (freeze/thawing). Since DSBs are one of the most important types of DNA damage and the results of our experiment demonstrated that DMSO could decrease both physically and chemically induced DSBs, DMSO should be very effective in protecting DNA molecules from DSBs. As past research demonstrated that high concentration (more than 10%) of DMSO exhibit various effects on biological systems, which was very toxic^28, 31, 37^. Thus, the results from our experiment suggested that ‘2% DMSO’ would provide a good starting point in future experiments in biological and medical sciences as well as cryopreservation.

In summary, the single-DNA observation provided useful kinetic data for quantitatively evaluating DSBs. Extension of such observation is expected for the measurements of DSBs caused by other types of injuries, such as mechanical stress^53^, ultrasound^16^, and radiations of heavy ion, proton, x-ray, etc.

## Materials and Methods

### Materials

T4 phage DNA (166 kbp) was purchased from Nippon Gene (Toyama, Japan). DMSO was obtained from Wako Pure Chemical Industries (Osaka, Japan). The fluorescent cyanine dye, YOYO-1 (quinolinium, 1,1′-[1,3-propanediyl-bis[(dimethylimino)-3,1-propanediyl]] bis[4-[(3-methyl-2(3H)-benzoxazolylidene)-methyl]]-tetraiodide), was purchased from Thermo Fisher Scientific Corporation (Waltham, MA). The antioxidants, 2-mercaptoethanol (2-ME), and other necessary chemicals, were purchased from Wako Pure Chemical Industries (Osaka, Japan). The concentration of DMSO is given in (v/v)% throughout the present manuscript.

### Real-time observation of photo-induced breakage

In fluorescence microscopic observations, measurements were conducted at a low DNA concentration (0.1 µM in nucleotide units). T4 phage DNA (final concentration 0.1 μM) was dissolved in a solution containing 0.05 μM YOYO-1. The antioxidant 2-ME (4 (v/v)%) was added to the samples to retard the photo-cleavage reaction so as to detect the reaction rate of DSB by real-time observation. YOYO-1 was used as a photosensitizer to generate ROS^42, 43^, and the real-time observation of DSBs was performed in individual DNA molecules, where YOYO-1 also helped to visualise DNA at a peak emission wavelength of 510 nm under light illumination at 450–490 nm. Fluorescence images of DNA molecules were captured by using an Axiovert 135 TV (Carl Zeiss, Jena, Germany) microscope equipped with an oil-immersed 100 × objective lens and were recorded on a DVD through an EBCCD camera (Hamamatsu Photonics, Hamamatsu, Japan). All observations were carried out at room temperature (24 °C)^15, 44^.

### Measurements of the contour length of DNA molecules to evaluate the injury caused by γ-ray and freezing

DNA solutions with different DMSO concentrations were irradiated with ^60^Co γ-ray at a dose rate of 28 Gy/min. The quantity of γ-rays was controlled by the duration of irradiation^19, 44^. For the evaluation of freezing-induced DSB, DNA samples in a DMSO-water solution were frozen to −25 °C (freezing speed: ca. −0.4 K/min) and −80 °C (freezing speed: ca. −0.9 K/min) for 4 hours with electric freezers. They were then thawed at 4.2 °C (NIHON FREEZER, Tokyo, Japan) for about 12 hours.

Just before the measurements by fluorescence microscopy, DNA molecules were stained with YOYO-1 (final concentration: 0.05 μM). Glasses were pre-treated with poly-(L-lysine) (concentration: 0.05 (v/v)%) solution, and washed repeatedly with distilled water. A droplet (10 μL) of a sample was absorbed on a modified glass slide and covered with a glass cover slide under weak shear. Fluorescence images were observed with an Axiovert 135TV microscope (Carl Zeiss, Jena, Germany) and analysed using ImageJ software (National Institute of Mental Health, MD, USA)^16, 44^.
